# A Multimodal Time Point Labeling Approach for Analyzing Mastication and Swallowing Dynamics

**DOI:** 10.3390/bios16050301

**Published:** 2026-05-21

**Authors:** Jingjing Liu, Yuxuan Cao, Jiale Kuang, Zhongren Wei, Boyu Liu, Xianghao Wu, Bolin Shi, Lei Zhao, Dongfu Xu, Xinyu Wang, Kui Zhong

**Affiliations:** 1 College of Automation Engineering, Northeast Electric Power University, Jilin City 132012, China; 2 Key Laboratory of Food Sensory Analysis, State Administration for Market Regulation, Beijing 102200, China; 3 Agriculture and Biotechnology Standardization Sub-Institute, China National Institute of Standardization, Beijing 102200, China

**Keywords:** electromyographic, laryngeal, PVDF flexible piezoelectric sensor, mastication, swallowing

## Abstract

Mastication and swallowing are complex physiological processes involving the coordinated activity of multiple tissues in the oral cavity, facial region, and laryngeal system. Some detection methods suffer from limitations such as insufficient information acquisition and inadequate temporal feature analysis. To address these issues, this study proposes a conceptual method for analyzing the state of masticatory and swallowing movements. It integrates maxillofacial electromyographic (EMG) signals with laryngeal movement signals. The goal is to preliminarily explore state analysis of masticatory and swallowing movements over time. A designed gain-adjustable conditioning circuit processes and acquires these signals: maxillofacial EMG signals from EMG electrodes and laryngeal movement signals from flexible PVDF piezoelectric sensors. These two signal streams complement each other’s missing information, enabling comprehensive detection of the state of masticatory and swallowing movements. To address time-point labeling in mastication and swallowing, a sliding-window-based dispersion calculation method was employed to extract characteristic signal nodes, which were then accurately associated with their corresponding physiological motion states. We combined temporal features such as the zero point, onset of fluctuations, characteristic peaks, and baseline recovery from electromyographic (EMG) signals and laryngeal movement signals. This allowed us to establish a correspondence between key time points in the mastication and swallowing processes. The coefficient of determination (R^2^) for the pressure–voltage linear fit of the PVDF flexible piezoelectric sensor was 0.99446. The pressure resolution was approximately 0.08 kPa. Response times were no more than 15 ms for the EMG channel and no more than 10 ms for the PVDF pressure channel. These results indicate that this method is feasible for extracting oral movement time parameters in healthy subjects.

## 1. Introduction

Mastication and swallowing are fundamental physiological processes underlying food intake, involving complex and coordinated interactions among oral structures and muscular tissues [1,2]. Physiologically, mastication relies on the periodic movement of the mandible and the coordinated contraction of the masticatory and lingual muscles to achieve comminution and bolus formation. Swallowing, in turn, is accomplished through a sequence of actions, including contraction of the suprahyoid muscles, vertical displacement of the thyroid cartilage, and relaxation of the pharyngeal sphincters, thereby enabling bolus propulsion and transit into the pharynx. The coordination and continuity between these two processes, as well as their motion states, directly affect the proper functioning of the oral system [3,4,5]. Once abnormalities occur in mastication or swallowing, they may lead to disorders such as dysphagia and temporomandibular joint dysfunction, which can severely impair nutritional intake and quality of life [6,7,8].

Analyzing the state of oral movements and the changes in key phases can provide a kinematic basis for the prevention of oral disorders and the diagnosis of diseases. However, current assessments of oral motor function lack detailed methods for delineating key time points during the mastication–swallowing process, and the single signals extracted in some studies suffer from insufficient information content. Therefore, establishing precise and efficient quantitative methods for investigating oral motor patterns is of great significance. Such methods can not only advance the field of oral healthcare but also provide valuable references for food texture design and the development of assistive devices for swallowing rehabilitation [8].

At present, a series of studies using various detection techniques have been conducted in the field of oral motor monitoring [9,10,11,12,13]. To investigate the time sequence and amplitude variations in muscle contractions during mastication and to elucidate motor control strategies, the Sanjani group employed electromyographic (EMG) electrodes placed on the masseter, temporalis, and mylohyoid muscles for signal acquisition [14]. However, EMG-based measurement of complete motor states typically requires the deployment of multiple surface and invasive sensing nodes. In addition, the inherent latency between EMG signals and actual motor actions may introduce deviations in practical measurements. In this context, the development of flexible mechanical sensing technologies, such as piezoelectric and pressure sensors, has provided new approaches for the mechanical characterization of oral motion [15,16,17].

Tongue pressure signals, as a class of direct indicators reflecting tongue–palate contact states, have been used to compensate for the limitations of EMG signals [18,19]. In a study by the Nagasaki group, pressure sensors combined with EMG sensors were employed to assess oral motor states in patients with mandibular prognathism, with the aim of distinguishing differences in the onset time, offset time, and duration of tongue and muscle activities between patients with masticatory disorders and healthy individuals [20]. Nevertheless, these methods still presented certain limitations. Some detection schemes rely on invasive designs, which impose operational constraints in both clinical and experimental applications. Moreover, sensor-human body compliance needs further improvement, making it difficult to achieve precise, stable, and comfortable acquisition of oral motor signals.

This study proposes a non-invasive, multi-sensor fusion method for capturing oral motion data. The method aims to identify key time points in mastication and swallowing and to track motion states over time. Single-sensor detection methods struggle to capture muscle, mandibular, and laryngeal movements simultaneously. Aligning signals across modalities and identifying motion events are still critical challenges in time-point extraction.

To address this, this study combines the detection of core muscle groups and laryngeal movement. EMG sensors capture contraction of the masseter and hyoid muscles, while laryngeal pressure sensors reflect movements of the thyroid cartilage and tongue. This yields complementary multimodal information. Aligning with the timing of mastication and swallowing, the study correlates muscle and laryngeal changes with dual-modal signal features to identify key events. The paper introduces a dual-channel oral movement system and a sliding-window temporal analysis method, and presents results on signal features and time-point extraction from healthy-subject experiments.

## 2. Non-Invasive Oral Motion Data Acquisition Method

### 2.1. Operating Principle of the Non-Invasive Information Acquisition System

**Figure 1:**
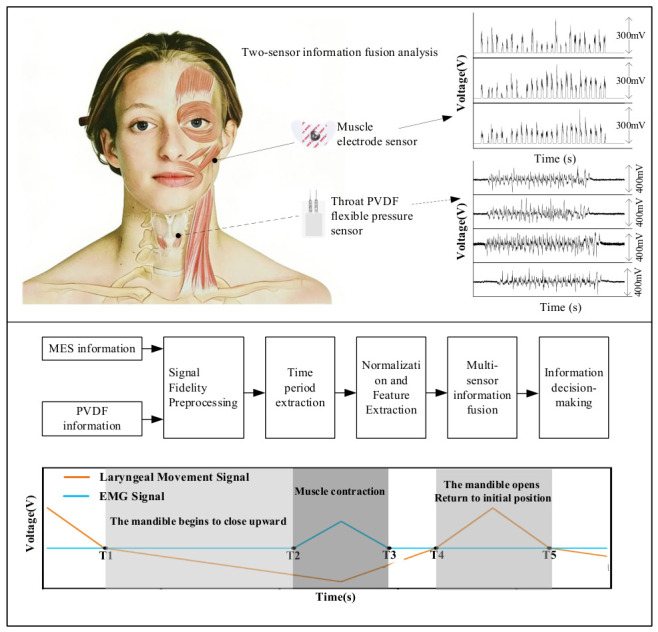
Schematic diagram of key temporal node calibration method for oral motor based on multi-signal fusion.

To obtain comprehensive oral motor information, the selection of measurement locations was considered a critical factor. According to relevant studies, laryngeal motion can integratively reflect the movement characteristics of the tongue muscles and mandible. When combined with electromyographic (EMG) signals to supplement contraction information from the masseter and suprahyoid muscles, it enables complete detection of mastication and swallowing activities [21]. Based on this rationale, the present study developed a mastication–swallowing motion detection system integrating EMG sensors and piezoelectric sensors, designed for monitoring maxillofacial muscle activity and the motion of the laryngeal thyroid cartilage. The overall design framework and signal processing workflow are illustrated in Figure 1. The core concept of the system was to acquire surface EMG signals and laryngeal mechanical pressure signals during mastication and swallowing using EMG electrodes and polyvinylidene fluoride (PVDF) flexible pressure sensors. Through a standardized processing pipeline—including signal preprocessing, effective time-segment extraction, signal normalization, feature extraction, and multi-sensor data fusion—the system enabled the identification of key time points in mastication and swallowing. In the signal processing procedure, filtering algorithms were first applied to eliminate common interference and high-frequency noise. Subsequently, a sliding-window-based dispersion calculation was employed to segment individual response intervals, thereby achieving time unification between the two modalities of signals. Finally, the key time points of mastication and swallowing were determined through the computation of relevant analytical formulations.

In the proposed detection system, sensing locations were selected based on the primary muscles involved in mastication and swallowing, with EMG sensors attached to the skin surface at the central region of the mandible. The monitored muscles include the masseter and the genioglossus–hyoid muscle group, which correspond to the principal muscle groups engaged in mastication and swallowing, respectively. Transient burst-like variations in EMG signals and laryngeal motion signals could accurately characterize oral motor states [22], enabling the extraction of muscle activity timing as well as onset and offset nodes. During mastication, occlusal contact between the upper and lower jaws triggers burst contractions of the masseter muscle, whose signal features can precisely indicate the onset, termination, and duration of muscle activity. During swallowing, the laryngeal thyroid cartilage undergoes displacement and deformation in response to the coordinated contraction and relaxation of associated muscle groups. For instance, the thyrohyoid muscle exhibits early activation associated with mandibular opening prior to the relaxation of the mylohyoid muscle [23,24]. Studies by Ko et al. indicate that surface electromyographic signals from the submental region can characterize features of the suprahyoid muscle group during swallowing, such as activation timing, activity duration, and peak amplitude [25]. Electrophysiological activity from the mylohyoid and geniohyoid, captured by EMG sensors, forms the basis for quantifying critical temporal milestones of swallowing: onset, peak contraction, and relaxation termination. Previous studies demonstrated functional differences between mandibular movement and suprahyoid muscle activity during mastication, while no significant differences have been observed between bilateral signals. Based on this finding, the present study simplifies sensor placement by acquiring signals from a single side only.

### 2.2. Preparation of the Sensor Unit for the Information Acquisition System

Accurate acquisition of laryngeal motion and EMG signals imposes stringent requirements on sensor sensitivity and conformability. To address this, a PVDF-based piezoelectric strain sensor (Zhimeikang Technology Shenzhen Co., Ltd., Shenzhen, China, 28 μm × 203 mm × 280 mm) and commercial EMG electrodes (Dukang Medical Devices Co., Ltd., Shijiazhuang, China, DK-01) were employed in this study. A PDMS (Dow Corning, Midland, MI, USA, SYLGARD 184) encapsulation layer was introduced to enhance stress transfer and improve the adhesion performance of the piezoelectric sensor. The PVDF sensor was fabricated using a sandwich-structured encapsulation. Specifically, the PDMS base and curing agent were mixed at a ratio of 10:1 and ultrasonically agitated for 30 min. Subsequently, 1 mL of the mixture was dispensed onto a glass substrate and spin-coated at 500 r/min for 10 s, followed by 1000 r/min for an additional 10 s. The film was then thermally cured at 100 °C for 30 min to obtain a PDMS layer. PVDF films of appropriate dimensions were prepared as the sensing element, with copper foil serving as the electrodes. The PVDF layer was encapsulated on both sides using the prepared PDMS films, resulting in a flexible PVDF-based piezoelectric pressure sensor [26].

For EMG signal acquisition, commercial surface EMG electrodes were utilized. Based on the non-invasive acquisition principle of surface EMG signals, this technique has been widely adopted in clinical medicine and human–machine interaction, and can accurately reflect the electrophysiological processes of muscle contraction and relaxation [27].

### 2.3. Dual-Channel Signal Acquisition Circuit Design

**Figure 2:**
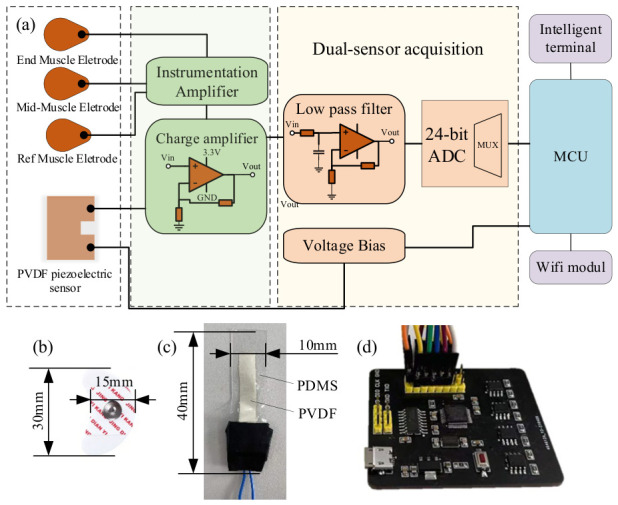
Circuit design diagram: (**a**) schematic diagram; (**b**) EMG electrode; (**c**) PVDF piezoelectric electrode; (**d**) circuit board.

A dual-channel synchronous signal acquisition circuit was developed to enable simultaneous acquisition of surface electromyographic (EMG) signals and laryngeal pressure or motion signals. The circuit was specifically designed to accommodate the unique characteristics of these target signals. This dual-channel configuration offers high sensitivity and signal adaptability, facilitating targeted conditioning and amplification of weak signals from both the PVDF sensor and surface EMG electrodes. As illustrated in Figure 2, the complete system comprises an analog front-end conditioning module, an ADS122C04 analog-to-digital converter (ADC), a microcontroller unit (MCU) control module, and a wireless data transmission module. Following independent front-end conditioning, the signals from both channels are input into the 24-bit ADC module, and the MCU manages synchronous acquisition, data integration, and transmission.

The EMG signal channel utilizes an instrumentation amplifier as the front-end differential amplification stage to extract weak differential signals from bipolar surface EMG electrodes and improve common-mode rejection. The front-end amplification stage provides a gain of approximately 11.51×, while the subsequent amplification circuit offers a gain of 50×. Consequently, the nominal total voltage gain of the EMG channel is approximately 575.5×, equivalent to about 55.2 dB. This level of gain amplifies weak surface EMG signals to a voltage range suitable for effective acquisition by the analog-to-digital converter (ADC).

The laryngeal pressure and motion signal channel utilizes a charge-amplification and signal-conditioning front end to convert the charge variations produced by the PVDF flexible piezoelectric sensor during laryngeal movement into an analog voltage signal suitable for data acquisition. This channel incorporates a charge amplification structure with a feedback capacitance of 1 nF and a feedback resistance of 1 GΩ. The resulting charge-to-voltage conversion gain is approximately 1 V/nC, with a low-frequency cutoff frequency near 0.16 Hz. The acquired laryngeal signal predominantly reflects low-frequency mechanical pressure changes induced by thyroid cartilage movement.

To mitigate 50 Hz power-line interference introduced by the AC power supply, a 50 Hz notch filter is incorporated into the EMG channel to reduce power-line noise in the experimental environment. The EMG signal is then processed using a 20–150 Hz bandpass filter in software to minimize low-frequency baseline drift, motion artifacts, and high-frequency noise during node extraction. As laryngeal pressure and motion signals primarily reflect low-frequency mechanical changes associated with thyroid cartilage movement, low-pass filtering and smoothing are applied to enhance the main motion waveform characteristics. The corresponding hardware circuit design is presented in Figure 2.

### 2.4. Performance Characteristics of the Oral Signal Acquisition System

To ensure the applicability of the device for oral motor information acquisition and the validity of the collected signals, four key performance metrics were evaluated: linearity, pressure resolution, response time, and repeatability. For linearity, the small-amplitude pressure and deformation changes resulting from the movement of the thyroid cartilage and adjacent laryngeal structures during swallowing were considered. The output voltage response was measured at various loading pressures within a low-load operating range, simulating conditions for laryngeal movement detection. A linear fit was applied to the pressure–voltage relationship. The coefficient of determination (R^2^) was 0.99446, demonstrating that the PVDF flexible piezoelectric sensor exhibits good dynamic response and a highly linear pressure–voltage relationship. The results showed a coefficient of determination of R^2^ = 0.99446, indicating an excellent linear response. For pressure resolution, gradient micro-pressure inputs were applied to determine the minimum detectable pressure increment that the sensor could reliably distinguish. The PVDF flexible piezoelectric sensor exhibited a pressure resolution of ≤0.08 kPa, enabling precise detection of subtle variations in muscle activity and laryngeal motion. For response time, trigger-based experiments were conducted to measure the time required for the sensor to produce a stable output signal after receiving a physiological stimulus. The results demonstrated that the response time was ≤15 ms for the maxillofacial EMG sensor and ≤10 ms for the PVDF flexible piezoelectric sensor. In terms of repeatability, the coefficient of variation for the test results was calculated from multiple repeated tests conducted under identical experimental conditions: ≤5% for EMG and ≤2% for PVDF [28]. All four performance metrics fall within ideal ranges, indicating that the device is suitable for analyzing oral motor signals. The corresponding results are summarized in Table 1.

## 3. Time-Series-Based Multimodal Data Fusion Method

To extract the time points corresponding to motion state transitions during mastication and swallowing, it is necessary to eliminate the temporal discrepancies among heterogeneous signals and achieve the matching of physiologically theoretical nodes with signal variation nodes. In this regard, this study investigated the physiological mechanism characteristics of mastication and swallowing, and proposed a node extraction theory for corresponding time points based on the signal variations in the two-modal data. Ultimately, it realized the time-resolved feature analysis and key node annotation for mastication and swallowing movements, respectively.

In the specific implementation, the signal processing methods for mastication and swallowing adopted a similar framework. First, the signal dispersion was calculated via a sliding window approach to segment the effective data intervals corresponding to a single mastication or swallowing event from the electromyographic (EMG) and laryngeal pressure signals. Subsequently, the signal extrema were used as matching parameters to achieve time unification and data integration of the two sensing modalities. Using this key time point extraction method, a total of five time points for mastication and eight time points for swallowing were identified. The relevant key parameters include dispersion and characteristic extrema, as defined in Equations (1) and (2), respectively.

1. Signal Dispersion

Dispersion is employed to characterize the fluctuation level of a signal sequence within a time window. When the human body is in a resting state, both EMG signals and laryngeal motion signals remain stable at baseline. During changes in mastication or swallowing activity, the signals exhibit significant fluctuations corresponding to the physiological movements, resulting in a sharp increase in dispersion. In this study, the signal dispersion *D* was defined as the ratio of the standard deviation to the mean of the signal sequence, as expressed in Equation (1):

(1) $$D=\frac{\sigma }{\mu }=\frac{\sqrt{\frac{1}{n}{\sum_{i=1}^{n}\left(x_{i}-\mu \right)}^{2}}}{\frac{1}{n}\sum_{i=1}^{n}x_{i}}$$

In the equation, *x*_*i*_ denotes the sampled signal value at the *i*-th time point, *n* represents the length of the signal sequence, *σ* is the standard deviation of the signal sequence, and *μ* is the mean of the signal sequence.

2. Signal Extremum

Within the effective signal acquisition interval [*t*_*s*_,*t*_*e*_], the maximum value of the EMG signal *EMG*_*max*_ and the extremum of the laryngeal motion signal (minimum value *P*_*min*_ for mastication, maximum value *P*_*max*_ for swallowing) were used as matching parameters for signal fusion. The calculation of these signal extrema is given in Equations (2)–(4):

(2) $$EMG_{\max}=\max\left\{EMG\left(t\right)|t_{s}\leq t\leq t_{e}\right\}$$

(3) $$P_{\min}=\min\left\{P\left(t\right)|t_{s}\leq t\leq t_{e}\right\}$$

(4) $$P_{\max}=\max\left\{P\left(t\right)|t_{s}\leq t\leq t_{e}\right\}$$

In the equation, *EMG*(*t*) denotes the sampled value of the electromyographic (EMG) signal at time *t*, and *P*(*t*) represents the sampled value of the laryngeal motion signal at time *t*.

### 3.1. Analysis of Mastication State Transition Nodes

Specifically, the mastication process proceeded sequentially through mandibular closure, muscle contraction, mandibular opening, and bolus aggregation. The synergistic relationship between EMG signals and laryngeal motion signals during mastication provided the fundamental basis for signal alignment and node identification [29,30,31,32]. By incorporating the physiological characteristics of mandibular opening–closing cycles and the contraction–relaxation patterns of the masseter muscle, the mastication process was decomposed into distinct state transitions. These state changes were then systematically mapped to the corresponding variation patterns in EMG and laryngeal motion signals, enabling accurate matching of mastication-related time points. Ultimately, five key time points (T1–T5) corresponding to the physiological process of mastication were extracted. The node extraction procedure first employed signal dispersion to determine the effective signal interval, followed by the use of characteristic extrema to unify the timescales of multimodal signals. Subsequently, precise annotation of the five identified time points was accomplished based on the zero-crossing points of the laryngeal motion signal, as well as the onset and return-to-baseline points of the EMG signal. The five identified time points comprehensively covered the entire physiological cycle of mastication, from initiation and occlusal contraction to bolus aggregation and the termination of the movement.

The extraction of effective signal segments was based on the dispersion parameter. The dispersion of the EMG signal *D*_*EMG*(*t*)_ and the laryngeal motion signal *D*_*P*(*t*)_, computed within sliding windows, is compared with a predefined dispersion threshold *D*_0_ to determine the start time *t*_*s*_ and end time *t*_*e*_ of the effective signal segments. Accordingly, the effective interval of the EMG signal was defined as *T*_*EMG*_ = [*t*_*EMGs*_,*t*_*EMGe*_], and the effective interval of the laryngeal motion signal was defined as *T*_*P*_ = [*t*_*Ps*_,*t*_*Pe*_]. After signal alignment based on the signal extrema, a common effective analysis interval was determined as *T* = [*t*_*s*_,*t*_*e*_], yielding a 2 × N dataset. The specific implementation is given in Equations (5)–(7):

(5) $$t_{EMG\max}\Leftrightarrow t_{P\min}$$

(6) $$t_{s}=max\left(t_{EMGs},t_{Ps}\right)$$

(7) $$t_{e}=min\left(t_{EMGe},t_{Pe}\right)$$

Here, *ts* is defined as the minimum onset time among the effective intervals of the two signal modalities, and *te* is defined as the maximum termination time. In this study, synchronized EMG and laryngeal motion signals from three consecutive mastication cycles were extracted, and the middle cycle was selected as the representative case for analysis.

**Figure 3:**
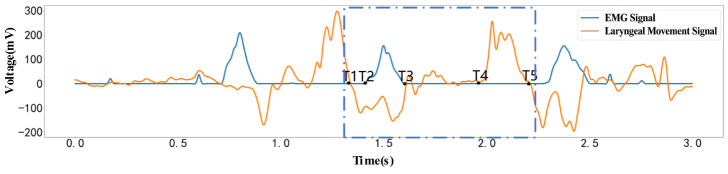
Time Points of the Mastication Process.

Based on the dispersion variations in the laryngeal motion signal and the EMG signal, five characteristic time points (T1–T5) were defined, and the extracted results are shown in Figure 3. The trend of mandibular movement can be represented by the variation in the laryngeal motion signal: when the mandible moves downward, the laryngeal pressure signal is positive, whereas when the mandible moves upward, the signal is negative.

T1 represents the transition between the conclusion of the previous mastication cycle and the initiation of the subsequent cycle. At this stage, the oral cavity is relatively relaxed with increased volume, reduced masseter muscle activity, and the mandible positioned either stably or slightly displaced downward. Under these physiological conditions, both the electromyographic (EMG) signal and the laryngeal movement signal return to near-baseline levels, indicating stabilization of the current response [29,33]. As illustrated in the figure, both signals approach baseline at T1, and the following T1–T2 interval signifies the onset of mandibular upward closure. Thus, T1 is defined as the transition point between the end of one masticatory cycle and the beginning of the next. The calculation for the T1 node is provided in Equation (8):

(8) $$T1=\left\{t\in \left(t_{s},t_{e}\right)|P\left(t\right)=0\cap \frac{dP(t)}{dt}<0\right\}$$

Beginning at T2, the electromyography (EMG) signal increases, indicating contraction of the masseter muscle. At this point, the upper and lower jaws are fully closed, and the tongue attains its most posterior position against the palate [34]. As mastication continues, the tongue’s cyclic trajectory gradually shifts upward, approaching the palate and incrementally moving the food forward. The calculation for the T2 node is presented in Equation (9):

(9) $$T2=\left\{t\in \left(t_{s},t_{e}\right)|D_{EMG\left(t\right)}\geq D_{0}\cap \frac{dEMG\left(t\right)}{dt}>0\right\}$$

T3 denotes the transitional phase in the masticatory cycle when the masseter muscle ceases exertion and adjusts the position of the food. During this phase, masseter muscle contraction activity decreases. These physiological changes result in a reduction in both the number of recruited motor units and their discharge activity, as demonstrated by a gradual decline in surface electromyography (EMG) amplitude [35]. As illustrated in the figure, the EMG signal at the T3 node drops significantly to near baseline, while the laryngeal movement signal remains predominantly in the negative half-space. This pattern indicates that the mandible has completed its upward closing movement or is in a brief stable state. The observed signal change corresponds with the physiological process of the masseter muscle ceasing contraction and entering the food repositioning phase. Therefore, T3 can be defined as the key time point in the masticatory cycle that marks the end of masseter contraction, the termination of the occlusal force phase, and the transition to the food repositioning phase. The calculation of the T3 node is given in Equation (10):

(10) $$T3=\left\{t\in \left(T2,t_{e}\right)|D_{EMG\left(t\right)}\geq D_{0}\cap \frac{dEMG\left(t\right)}{dt}<0\right\}$$

T4 represents the phase of the masticatory cycle during which occlusal force ceases and the mandible initiates opening, while the tongue contributes to food positioning and gathering. In this phase, masseter muscle contraction diminishes, contact between the teeth and food is temporarily interrupted, and the mandible opens. The tongue subsequently aids in gathering and positioning the food, thereby controlling bolus formation. These physiological changes result in deformation of tissues associated with the larynx, neck, and hyoid bone, which is reflected as a convex waveform in the laryngeal movement signal [4,36]. As illustrated in the figure, the electromyographic signal at T4 returns to near baseline, whereas the laryngeal movement signal demonstrates an upward fluctuation. This indicates a shift in the primary signal characteristic from masseter muscle activity to laryngeal movement response. This signal transition corresponds to mandibular opening and food repositioning; thus, T4 is defined as the key time point marking the onset of mandibular opening and bolus repositioning within the masticatory cycle. The calculation of the T4 node is given in Equation (11):

(11) $$T4=\left\{t\in \left(T1,t_{e}\right)|D_{P\left(t\right)}\geq D_{0}\cap \frac{dP(t)}{dt}>0\right\}$$

T5 represents the stage in the masticatory cycle when mandibular opening and food repositioning are nearly complete. During this phase, mandibular opening concludes gradually, while the tongue positions and gathers food particles in preparation for the subsequent occlusal movement. Throughout these physiological processes, masseter muscle contraction remains minimal, and responses to soft-tissue stretching in the larynx and anterior neck progressively stabilize [37]. As illustrated in the figure, electromyographic signals at the T5 time point remain near baseline, and laryngeal movement signals have also decreased, indicating that the primary laryngeal movement response during this mastication cycle is nearing its conclusion. Thus, T5 can be identified as a key time point marking the end of the current mastication cycle and the onset of the preparation phase for the next occlusion. The calculation of the T5 node is given in Equation (12):

(12) $$T5=\left\{t\in \left(T4,t_{e}\right)|D_{P\left(t\right)}\geq D_{0}\cap \frac{dP(T_{5})}{dt}<0\right\}$$

### 3.2. Analysis of State Change Nodes During Swallowing Motion

Similar to the analytical framework for mastication, the time unification of EMG signals and laryngeal motion signals in the swallowing process was grounded in the physiological coupling between neuromuscular contractions and laryngeal mechanical movements during swallowing [32,38]. The swallowing process primarily consists of two stages: bolus transport and bolus entry into the pharynx. The implementation followed a procedure analogous to that used for mastication signal processing. By incorporating the physiological characteristics of multi-stage contraction and relaxation of swallowing-related muscle groups, as well as the regular displacement of the thyroid cartilage, the key states of swallowing were systematically matched with variations in EMG and laryngeal motion signals. Ultimately, eight key time points (T6–T13) corresponding to the physiological swallowing process were extracted. In the implementation, a sliding-window approach was applied to calculate the dispersion of the EMG signal *D*_*EMG*_(*t*) and the laryngeal motion signal *D*_*P*_(*t*), respectively. Based on a unified dispersion threshold *D*_0_, the variations in dispersion for both signal types were evaluated. The onset time, termination time, and all sampled signal points within each interval were synchronously recorded, thereby capturing the effective EMG signal interval *T*_*EMG*_ = [*t*_*EMGs*_,*t*_*EMGe*_] and the effective laryngeal motion signal interval *T*_*P*_ = [*t*_*Ps*_,*t*_*Pe*_]. The specific segmentation rules are defined in Equations (13)–(15):

(13) $$t_{EMG\max}\Leftrightarrow t_{P\max}$$

(14) $$t_{s}=max\left(t_{EMGs},t_{Ps}\right)$$

(15) $$t_{e}=min\left(t_{EMGe},t_{Pe}\right)$$

Here, *ts* is defined as the minimum onset time among the effective intervals of the two signal modalities, and *te* is defined as the maximum termination time. After alignment, the analysis range of both signals was restricted to the common effective interval *T* = [*t*_*s*_,*t*_*e*_], thereby achieving time unification between the two modalities. This ensured that all sampled data points within the interval correspond to a single complete swallowing event.

**Figure 4:**
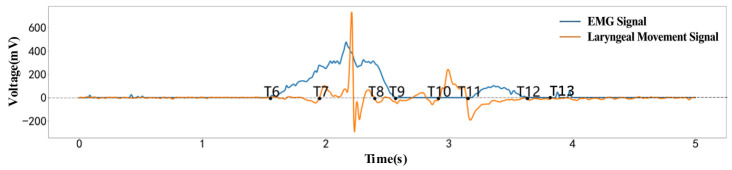
Time Points of the Swallowing Process.

Based on the characteristic variations in the zero-crossing points in the laryngeal motion signal, as well as the onset and return-to-baseline points of the EMG signal, eight key time points (T6–T13) were defined, as illustrated in Figure 4. The actual segmentation results were also shown in Figure 4.

T6 represents the transition from the bolus organization phase following mastication to the bolus transport phase. During this stage, the suprahyoid muscle groups, including the mylohyoid, anterior belly of the digastric, and genioglossus, begin to activate. This activation provides muscular support for tongue retraction and subsequent movements of the tongue, hyoid and laryngeal structures. These physiological processes result in the activation of swallowing-related muscle groups, as evidenced by electromyographic (EMG) signals that deviate from baseline and exhibit sustained fluctuations [34,39]. As illustrated in the figure, the EMG signal at T6 continues to rise, while the laryngeal movement signal fluctuates slightly around zero. This observation suggests that T6 primarily reflects preparatory activity in the swallowing-related muscle groups, with significant displacement of the thyroid cartilage and laryngeal structures yet to be initiated. Thus, T6 can be defined as the key time point marking the onset of activation in the swallowing-related muscle groups and the transition from the oral preparation phase to the bolus transport phase.

(16) $$T6=t_{s}$$

T7 represents the stage of swallowing during which the bolus-propulsion pathway gradually develops. Following the transition phase, intensified contraction of the suprahyoid muscle group and contact between the anterior tongue and hard palate establish the bolus propulsion pathway. These coordinated actions displace the thyroid cartilage and adjacent laryngeal structures, resulting in a significant increase in laryngeal movement signals [40]. As illustrated in the figure, the electromyographic signal at T7 continues to rise, and the initial distinct fluctuation in laryngeal movement signals emerges, reflecting sustained activity of swallowing-related muscle groups and the onset of laryngeal movement responses. Thus, T7 is defined as the critical time point during swallowing when laryngeal movement initiates and the bolus-propulsion channel forms.

(17) $$T7=t_{EMGs}=\left\{t\in \left(t_{s},t_{e}\right)|D_{P\left(t\right)}\geq D_{0}\right\}$$

T8 represents the transition from the early swallowing phase, during which the initial laryngeal movement nears completion, to the phase where the bolus is propelled into the pharynx. Between T7 and T8, the tongue body maintains contact with the hard palate, while the posterior tongue continues to move, advancing the bolus along the hard palate toward the oropharynx. As the backward propulsion of the bolus concludes, the larynx’s initial displacement response reaches a characteristic inflection point. The pressure on the PVDF sensor subsequently decreases, and the laryngeal movement signal trends toward the end of the initial wave [41]. At T8, the laryngeal movement signal shifts from a distinct wave to a decline phase, whereas the electromyographic signal remains at high amplitude. This pattern suggests that swallowing-related muscle groups continue to propel the bolus, although the larynx’s first-phase response is essentially complete. Thus, T8 serves as a characteristic time point that marks the end of the larynx’s initial mechanical response during early swallowing and the transition to the pharyngeal phase.

(18) $$T8=\left\{t\in \left(T7,t_{e}\right)|D_{P\left(t\right)}\geq D_{0}\cap P^{\prime }\left(t\right)<0\right\}$$

T9 represents the transition from the completion of the initial phase of muscle activity during the early bolus transport stage of swallowing to the subsequent pharyngeal entry stage. In the preparatory phase, muscle groups involved in swallowing, such as the suprahyoid muscles, coordinate to generate the primary contractions necessary for early bolus transport, while tongue movements propel the bolus toward the pharyngeal entrance. These physiological processes result in a gradual decline of the electromyographic signal from a sustained high amplitude to near baseline levels [42]. As illustrated in the figure, the first major electromyographic waveform terminates at T9, and laryngeal movement signals display only minor fluctuations after T8. This pattern indicates a progressive reduction in both the first-phase activation of swallowing-related muscle groups and the laryngeal response. Therefore, T9 is defined as the critical time point that signifies the end of the first segment of electromyographic activity during early bolus transport and the transition to the pharyngeal entry phase.

(19) $$T9=\left\{t\in \left(T8,t_{e}\right)|D_{EMG\left(t\right)}\geq D_{0}\cap {EMG}^{\prime }\left(t\right)<0\right\}$$

T10 represents the phase in the swallowing process when the bolus transitions from early transport to late preparation for pharyngeal entry. During this stage, activity in the primary swallowing muscle groups decreases, the bolus gradually approaches the entrance to the pharyngeal cavity, and the laryngeal structures begin movements associated with pharyngeal entry and airway protection. These physiological changes result in pressure alterations within the thyroid cartilage and adjacent laryngeal structures, leading to a second distinct increase in laryngeal movement signals [43]. As illustrated in the figure, the electromyographic signal at T10 remains near baseline, whereas the laryngeal movement signal demonstrates a second distinct fluctuation. This observation suggests that T10 primarily reflects the initiation of the laryngeal movement response during the late phase of swallowing. Therefore, T10 can be defined as the critical time point marking the onset of the secondary mechanical response of the larynx in the late phase of swallowing and the beginning of bolus preparation for pharyngeal entry.

(20) $$T10=\left\{t\in \left(T9,t_{e}\right)|D_{P\left(t\right)}\geq D_{0}\cap P^{\prime }\left(t\right)>0\right\}$$

T11 represents the late swallowing phase, during which the bolus enters the pharyngeal cavity for transport. At this stage, the base of the tongue moves backward and upward, and the soft palate elevates to protect the airway. The direction of laryngeal movement and local force conditions also change; swallowing-related muscle groups become active again, and the laryngeal signal shifts from a secondary rise to a negative region, accompanied by a secondary fluctuation in the electromyographic (EMG) signal [43]. As illustrated in the figure, laryngeal movement signals transition to a negative phase near T11, while the EMG waveform rises, indicating synchronous changes between laryngeal movement and activation of the swallowing muscle groups. Thus, T11 is defined as a key characteristic point in the late swallowing phase, determined by the transition to the negative phase of laryngeal movement and the synchronous rise in the second segment of EMG activity.

(21) $$T11=\left\{t\in \left(T9,t_{e}\right)|D_{EMG\left(t\right)}\geq D_{0}\cap {EMG}^{\prime }\left(t\right)>0\right\}$$

T12 and T13 represent the recovery phase in the late stage of swallowing, during which laryngeal movement and swallowing-related muscle activity progressively return to the resting state. Following T11, the pressure response of the thyroid cartilage and laryngeal structures diminishes, and the activation level of swallowing-related muscles decreases, as indicated by the return of both the laryngeal movement signal and the EMG signal to baseline levels [44]. The figure demonstrates that the laryngeal movement signal returns to near baseline at T12, indicating that the laryngeal response is nearing completion. Subsequently, the EMG signal returns to near baseline at T13, suggesting that the primary electrical activity of the swallowing-related muscles is also concluding. Thus, T12 and T13 serve as characteristic time points that mark the termination of laryngeal mechanical movement and EMG activity during the recovery phase of swallowing.

(22) $$T12=\left\{t\in \left(T11,t_{e}\right)|D_{EMG\left(t\right)}\geq D_{0}\cap {EMG}^{\prime }\left(t\right)<0\right\}$$

(23) $$T13=\left\{t\in \left(T10,t_{e}\right)|D_{P\left(t\right)}\geq D_{0}\cap P^{\prime }\left(t\right)>0\right\}$$

The time point extraction method developed in this section employs signal dispersion and characteristic extrema as key parameters to distinguish motion states and achieve alignment of multisource data. Based on this framework, the characteristic time points corresponding to the full mastication cycle and the multi-stage swallowing process are determined. Each identified time point is explicitly associated with specific physiological events, including mandibular opening and closing, muscle contraction, bolus transport, and airway protection. This approach enables precise annotation of key time points in oral motor activity.

## 4. Human Oral Motor Function Assessment

To demonstrate the practicality of the proposed method, signal acquisition experiments on mastication and swallowing were conducted in a healthy population. Based on the data obtained from the established noninvasive oral motion detection system, and using the proposed time point identification method for mastication and swallowing, a complete workflow was implemented, covering the entire process from data acquisition to time point extraction. To ensure a clearer distinction between the presentation of results and their physiological interpretation, the experimental signal acquisition process was clearly defined. The results section first describes the bimodal signal waveforms, effective movement periods, and extracted time points, followed by an explanation of the kinematic significance of each time point in conjunction with the physiological processes of chewing and swallowing.

### 4.1. Human Oral Motor Signal Acquisition Process

**Figure 5:**
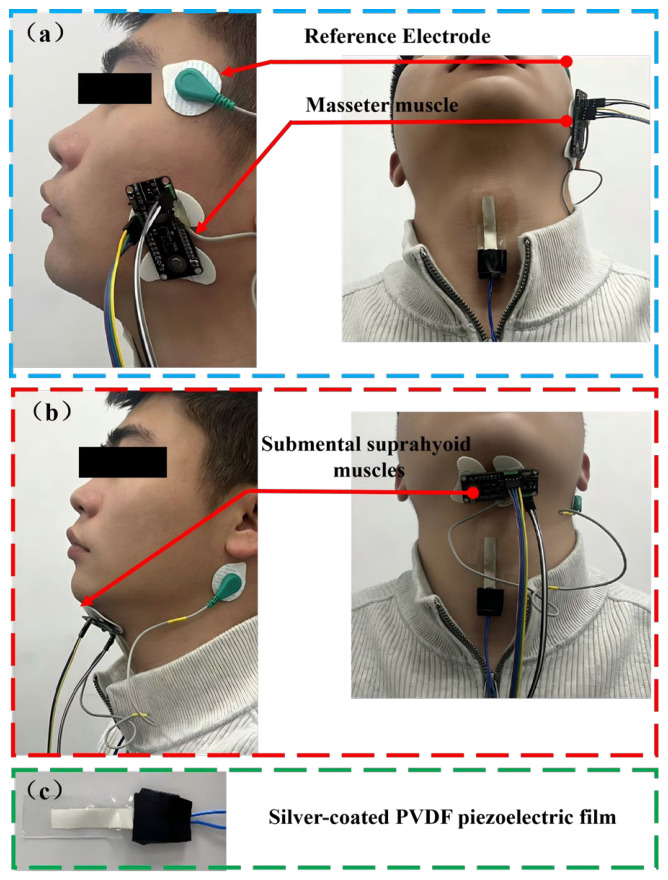
Sensor placement for laryngeal motion signal detection. (**a**) Sensor placement during the mastication experiment. (**b**) Sensor placement during the swallowing experiment. (**c**) A 1 cm × 4 cm flexible PVDF piezoelectric sensor.

The attachment positions and distribution of the two types of sensors are shown in Figure 5. Based on the characteristics of the target muscle groups and laryngeal structural movements during mastication and swallowing tasks, an individualized electrode/sensor placement strategy was adopted using anatomical landmarks, manual palpation, and motion-evoked signal confirmation.

During the mastication experiment, EMG electrodes were placed on the masseter muscle belly. Prior to placement, subjects were instructed to lightly clench or bite down on their teeth, and the area of the most prominent muscle belly elevation during masseter contraction was identified through palpation. The center of the electrode was placed within the superficial projection area of the masseter muscle, located between the inferior border of the zygomatic arch and the anterosuperior aspect of the mandibular angle, while avoiding areas near the tendon insertion as much as possible. The two recording leads of the bipolar electrode were arranged along the direction of the masseter muscle fibers—that is, roughly from the zygomatic arch to the mandibular angle—to enhance the stability of EMG signal acquisition related to masseter muscle contraction [45].

During the swallowing experiment, electromyography (EMG) electrodes were placed on the hyoid muscle group’s surface projection area in the submental region. Electrode placement was determined using anatomical landmarks: the inferior border of the mandible, the hyoid region, and the midline of the anterior neck. The electrode center was located approximately 15 mm lateral to the midline, between the inferior-posterior aspect of the mandible and the superior aspect of the hyoid bone. This area primarily reflects the activity of swallowing-related muscles, including the genioglossus, hyoglossus, and anterior belly of the digastric. EMG signal stability was verified by instructing participants to retract the tongue against the palate or to perform a slight swallowing movement [46].

The PVDF laryngeal motion sensor is positioned along the midline of the anterior neck, directly over the region of thyroid cartilage movement, ensuring that its sensitive area encompasses the full trajectory of the cartilage from rest to its maximal elevation during swallowing. This configuration enables the sensor to detect pressure, bending, and deformation signals produced by the movement of the thyroid cartilage and adjacent laryngeal structures throughout swallowing.

Prior to electrode application, the skin is cleansed using a single, brief wipe with 75% medical alcohol to remove surface oils and dead skin cells, thereby promoting optimal electrode-skin contact. Following electrode placement, signal stability is assessed both at rest and during specific movements, including gentle biting, tongue retraction against the palate, and test swallowing, to confirm secure and reliable electrode attachment.

All participants were healthy male adults aged 21–28 years, with a mean height of 175.5 ± 3.4 cm and a mean body weight of 76.2 ± 9.5 kg. None of the subjects had laryngeal diseases, oral or dental abnormalities, orthodontic treatment history, or temporomandibular joint disorders (TMD). All subjects were informed of the experimental procedures and materials and provided written informed consent. The research protocol was approved by the Ethics Committee of Northeast Electric Power University.

Before the formal experiment, subjects were asked to wear an eye mask to avoid the influence of visual cues. Each subject sat upright on a chair with the head slightly raised to prevent bending of the pressure sensor. The top of the sensor was aligned with the highest point reached by the thyroid cartilage prominence during swallowing, and the firm attachment of the EMG sensors was verified. After sensor placement, the subject slightly tucked the chin to keep the Frankfurt plane parallel to the ground. A disposable syringe was used to place 10 mL of warm water in the floor of the mouth. The subject was instructed to swallow upon receiving a start signal. After sensor attachment, each subject performed five swallowing tasks at 1 min intervals, completing the swallowing experiment. In the mastication experiment, each subject was provided with 4 g of chewing gum and instructed to chew continuously until the gum became soft. Once softened, the subject was asked to chew the gum ten consecutive times, and valid signals detected by the sensors during the ten mastication cycles were recorded. A total of 90 valid motor events were analyzed from six participants, comprising 30 swallowing events (six participants multiplied by five swallows) and 60 mastication cycles (six participants multiplied by ten chews). Swallowing was assessed as single, complete swallowing movements, whereas mastication was evaluated as individual mastication cycles.

### 4.2. Extraction of Time Points in Oral Motor Function

**Figure 6:**
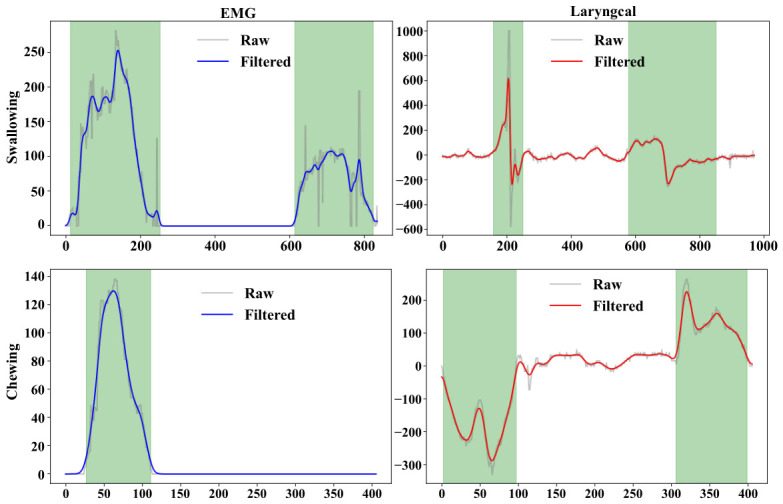
Dual-Sensor Signal Waveforms and Time Point Boundary Annotations.

To ensure accurate extraction of time points and to enable the identification of mastication and swallowing states based on these nodes, this section applies the proposed time point extraction method to capture key time features and corresponding time points of oral motor activities. Figure 6 illustrates the data processing workflow for both swallowing and mastication, including the signal waveforms and the annotated time points, which are generated by an algorithm developed based on the proposed method. From the perspective of signal characteristics, the filtered outputs of both sensors (with the EMG signal shown as the blue curve and the laryngeal motion signal as the red curve) clearly exhibited the typical physiological waveforms associated with swallowing and mastication. Specifically, the rising edge of the EMG signal corresponds to the initiation of contraction in the maxillofacial muscle groups, the peak represents the maximum contraction intensity, and the falling edge corresponds to the termination of muscle relaxation.

The characteristic fluctuations of the laryngeal motion signal correspond to the displacement process of the thyroid cartilage. The regions marked by the green shaded areas delineate the effective activity intervals of the motion. In the swallowing process, the first fluctuation region corresponds to the phase from swallowing initiation to bolus propulsion. During this stage, the EMG signal exhibits sustained high-amplitude variations with a single-peak or multi-peak pattern, while the laryngeal motion signal simultaneously presents a characteristic double-peak waveform. The time overlap between these two signals precisely corresponded to the physiological phase characterized by strong muscle contraction and significant upward displacement of the thyroid cartilage. The second fluctuation region corresponded to the late stage of swallowing, during which the bolus enters the esophagus and the muscle groups undergo relaxation; both the EMG and laryngeal pressure signals exhibit synchronized rise and decay in amplitude. In contrast, during mastication, the laryngeal motion signal exhibited a pattern of initial decrease followed by an increase, reflecting the dynamic movement of the mandible and tongue. The EMG signal, meanwhile, showed a pronounced single-peak waveform corresponding to the contraction of the masseter muscle.

**Figure 7:**
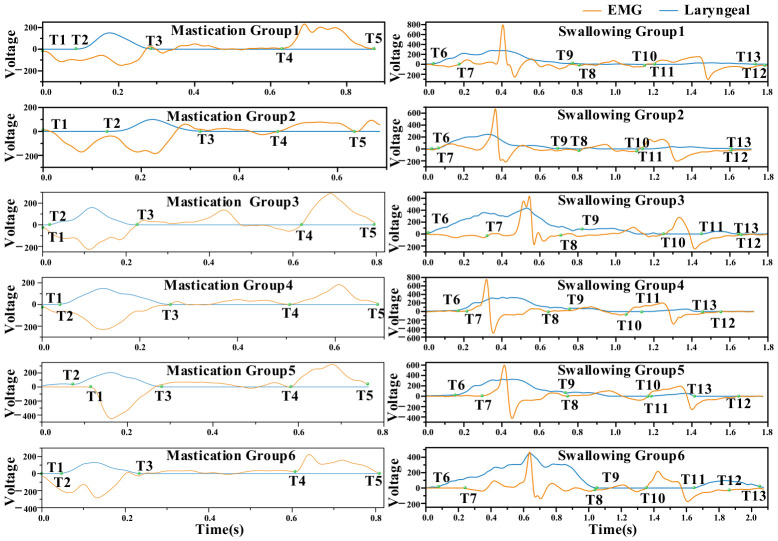
Temporal node calibration for mastication and swallowing in six subjects.

Figure 7 presents the results for different subjects. As observed, there were significant inter-individual differences in physiological signal response characteristics. However, with respect to mastication, a consistent pattern could still be identified. Specifically, T1 denotes the onset of the mastication cycle, during which the mandible moves upward, corresponding to a downward fluctuation in the laryngeal signal. T2 represents the initiation of masseter muscle contraction, at which point the EMG signal begins to increase. The interval from T1 to T2 corresponds to the transition from oral closure to the onset of masseter contraction and food grinding, with the tongue assisting in food propulsion. T3 marks the end of masseter relaxation, corresponding to the termination of EMG signal fluctuations and the complete relaxation of the muscle. The interval from T2 to T3 thus represents a complete contraction–relaxation cycle of the masseter muscle. T4 indicates the point at which the mandible completes its upward movement and begins to open. This stage is accompanied by an expansion of oral cavity volume, during which the buccinator muscle contracts to push the food back to the molar region, while the tongue intermittently performs reverse torsion to transfer the food to the contralateral occlusal surface. Finally, the mastication cycle concludes at T5. The interval from T4 to T5 corresponds to mouth opening and food transfer, preparing for the subsequent occlusal action. All subjects exhibited this continuous sequence of movements, indicating a consistent paradigm of mastication. This analytical result was also consistent with the time distribution observed in actual mastication behavior. The complete mastication process in humans is formed by the superposition of such repetitive motion cycles [47]. From the time distribution of each node, the time points extracted by the proposed method were in good agreement with the physiological characteristics of human mastication.

The time distribution of swallowing nodes is illustrated in Figure 7. It can be observed that, although there are inter-subject variations in signal amplitude, all subjects exhibited a similar swallowing motor pattern. This pattern can be divided into two stages: the bolus transport stage (T6–T9) and the swallowing stage (T10–T13). During the bolus transport stage, T6 represents the initiation point, corresponding to the onset of EMG activity in the mylohyoid muscle, anterior belly of the digastric muscle, and geniohyoid muscle, while the thyroid cartilage begins to prepare for movement. T7 marks the initiation of thyroid cartilage motion, corresponding to the first fluctuation in the laryngeal motion signal, indicating the onset of its downward displacement. The interval from T6 to T7 thus represents the initiation phase of bolus transport. T8 and T9 correspond to the endpoints of the first fluctuation in the laryngeal motion signal and EMG signal, respectively, with their time positions being closely aligned. During the interval from T7 to T9, the tongue dorsum elevates sequentially from anterior to posterior, propelling the bolus toward the entrance of the pharynx. The pharyngeal swallowing stage begins at T10 and T11, which are temporally proximate and correspond to the onset of the second fluctuation in the laryngeal motion signal and EMG signal, respectively. This stage represents a brief plateau following the elevation of the laryngeal prominence to its highest position, during which the bolus is about to enter the pharyngeal cavity. The tongue exerts a posterior-inferior force, coordinated with the contraction of pharyngeal muscles, to rapidly propel the bolus into the esophageal inlet. Simultaneously, the elevation of the tongue root indirectly compresses the epiglottis, causing it to cover the tracheal opening and thereby ensuring airway protection. T12 and T13 denote the termination points of the laryngeal motion signal and EMG signal, respectively. During the interval from T10 to T13, the mylohyoid muscle, the anterior belly of the digastric muscle, and the geniohyoid muscle complete the swallowing process. At this point, the entire swallowing cycle is concluded. These analytical results were consistent with the temporal distribution characteristics of human swallowing reported by the Tsukasa research group [20,44].

Figure 6 and Figure 7 display the dual-modal signal waveforms, the identified effective movement periods, and the extracted T1–T13 key temporal nodes. The experimental results demonstrate that the EMG signals and laryngeal movement signals exhibit strong temporal correspondence. While differences in signal amplitude were observed among subjects, the primary temporal movement patterns remained consistent. The physiological significance of the nodes was interpreted in relation to the mastication and swallowing processes. The extracted T1–T5 mastication nodes and T6–T13 swallowing nodes aligned with established human physiological movement patterns and previous research findings, supporting the use of this method for temporal node segmentation and state recognition during mastication and swallowing.

## 5. Conclusions

To analyze the time variations in mastication and swallowing states, this study proposed a noninvasive method for identifying key time points based on the fusion of maxillofacial EMG signals and laryngeal motion signals. Performance test results indicate that the dual-channel signal acquisition module designed in this study can rapidly acquire electromyography (EMG) and laryngeal pressure data with high linearity during mastication and swallowing movements. By integrating physiological movement theories of mastication and swallowing and by analyzing the acquired data for signal alignment and dispersion parameters, key time points were calibrated, thereby enabling the identification and classification of specific oral movement states. In assessing oral movements in healthy individuals, this study presents the experimental workflow for the signal-timing node calibration method. Based on mastication and swallowing signals collected from six participants, the results of the node-annotation algorithm are presented, along with the primary waveform characteristics of mastication and swallowing movements. Furthermore, the calibration process and corresponding movement phases for the five mastication movements and eight swallowing movements in relation to their physiological timing nodes are elaborated upon. In summary, as a proof-of-concept study, this research preliminarily validated the feasibility of calibrating mastication–swallowing time points based on the fusion of maxillofacial electromyographic (EMG) signals and laryngeal movement signals. Future studies will expand the sample size to include individuals with swallowing disorders or abnormal oral motor function, and will further validate the accuracy and clinical applicability of the calibration results by integrating independent reference standards such as video recordings and swallowing videofluoroscopy.

## Figures and Tables

**Table 1:** Device Performance Parameters.

Performance Index	Linearity	Precision	Response Time	Repeatability
Value	0.99446	PVDF piezoelectric sensor ≤ 0.08 kPa	EMG ≤ 15 ms PVDF ≤ 10 ms	EMG ≤ 5%, PVDF ≤ 2%

## Data Availability

The raw data supporting the conclusions of this article will be made available by the authors on request.
